# The evolution of domestic violence prevention and control in Vietnam from 2003 to 2018: a case study of policy development and implementation within the health system

**DOI:** 10.1186/s13033-019-0295-6

**Published:** 2019-06-08

**Authors:** Thi Minh Le, Christine Morley, Peter S. Hill, Quyen Tu Bui, Michael P. Dunne

**Affiliations:** 1 Dept. Population and Reproductive Health, Faculty of Health Social Sciences, Behaviour and Health Education, Hanoi University of Public Health, 1A Duc Thang Road, Duc Thang Ward, North Tu Liem District, Hanoi, Vietnam; 2 School of Public Health and Social Work, Queensland University of Technology, Brisbane, Australia; 3 School of Public Health, University of Queensland, Brisbane, Australia; 4 Faculty of Fundamental Science, Hanoi University of Public Health, 1A Duc Thang Road, Duc Thang Ward, North Tu Liem District, Hanoi, Vietnam; 5 Institute for Community Health Research, Hue University, Hue, Vietnam

**Keywords:** Domestic violence, Gender, Case study, Policy, Development, Implementation, Vietnam, Health system

## Abstract

**Background:**

Internationally, mental health and social care systems face significant challenges when implementing policy to prevent and respond to domestic violence (DV). This paper reviews the policy process pertaining to the national law on domestic violence prevention and control (DVPC) within the health system in Vietnam from 2003 to 2018, and critically examines the policy-making process and content, the involvement of key actors and the barriers to implementation within the health system.

**Methods:**

63 policy documents, 36 key informant interviews and 4 focus group discussions were conducted in Hanoi city, Bac Giang and Hai Duong provinces. The policy triangle framework was used to analyse the development and implementation process of the Law on DVPC.

**Results:**

The Vietnamese government developed the law on DVPC in response to the Millennium Development Goals reporting requirements. The development was a top–down process directed by state bodies, but it was the first time that international agencies and civil society groups had been involved in the health policy development process. The major themes that emerged in the analysis include: policy content, policymaking and implementation processes, the nature of actors’ involvement, contexts, and mechanisms for policy implementation. Policy implementation was slow and delayed due to implementation being optional, decentralization, socio-cultural factors related especially to sensitivity, insufficient budgets, and insufficient cooperation between various actors within the health system and other related DV support systems.

**Conclusion:**

The initial development process for DVPC Law in Vietnam was pressured by external and internal demands, but the subsequent implementation within the health system experienced protracted delays. It is recommended that the policy be revised to emphasise a rights-based approach. Implementation would be more effective if monitoring and evaluation mechanisms are improved, the quality of training for health workers is enhanced, and cooperation between the health sector and related actors in the community is required and becomes routine in daily work.

**Electronic supplementary material:**

The online version of this article (10.1186/s13033-019-0295-6) contains supplementary material, which is available to authorized users.

## Background

Domestic violence (DV) is a major global health issue. According to the World Health Organization (WHO) up to 71% of women aged between 18 and 60 who have ever been in a relationship have experienced emotional, physical or sexual violence, and between 15 and 29% of women globally have experienced multiple forms of abuse [1].

In Vietnam, the gravity of domestic violence was illustrated by a national baseline survey with 4838 women age from 18 to 60 years. This study by the General Statistical Office in 2010 [2] indicated a high prevalence of domestic violence, with more than half (58%) of adult females reporting experience of at least one type of DV. One-third (34%) of married women reported either physical or sexual violence from their husband at some time in their lives. One in every seventeen women (6%) reported physical violence in the year before the survey. The study also found that women were three times more likely to be abused by their husband than by another person [2]. That national study also found that approximately half of survivors (49%) never told anybody about their experience, with the confidential survey being the first and possibly only point of disclosure. Up to 87% of survivors had never accessed formal services for support, unless the (physical) violence was severe [2].

Domestic violence has been a focus of attention for international agencies around the world. Recognising DV as a human rights violation [3–5] the Vietnam government has issued a number of laws, regulations and legal documents in order to fulfil the nation’s gender equity commitment to the UN Millennium Development Goals (MDGs) [6]. The first law on domestic violence prevention and control (DVPC) was approved in 2007 and came into effect in early 2008. This legal reform helped to draw DV out of the private domain in Vietnam and into the attention of the general public and professional services. Specific guidelines for implementation of this law within the health system were introduced in 2009 and revised in 2017.

Although the law on DVPC has been established for more than 10 years, there are many challenges associated with administration and implementation. This study aims to describe the evolution process of the law on DV prevention and control in Vietnam. It explores the factors underpin the policy process, and the gaps between the development of laws and their implementation within the health system. This paper explores the factors that underpin the development of appropriate mental health policies and their effective implementation. The study sought to uncover what works well during the current DV law implementation, what does not work, and why. The study also considers changes that might lead to improved outcomes in order to meet Vietnam’s current Sustainable Development Goals commitments related to interpersonal violence.

## Methods

A case study design was applied. This design enables in-depth investigation of a phenomenon in a particular setting utilising multiple sources of evidence. Case studies are widely used in policy analysis [7] and because policy is usually embedded within complicated contexts, in specific institutions and can be interpreted differently by various people involved in the policy process [8].

In this study, the ‘case’ is the law on DV prevention and control. The main focus is the development and implementation of DV law within the context of the Vietnamese health system. The study sites included Hanoi capital city (national level) and two provinces (Bac Giang and Hai Duong). In each province, one district and one commune health centre were selected. Thirty-six in-depth interviews (IDIs) and four focus group discussions (FGDs) with a total of 57 participants were conducted. Data collection occurred between September 2017 and May 2018.

### Participants

Key informants (policy actors) for IDIs and FGDs were purposively selected with 57 participants identified as being directly involved in DVPC within the health system (health managers, doctors, nurses), and related actors such as international donors (UNFPA gender specialist), project officers, women’s union group and local authorities in the community. All were Vietnamese. The characteristics of the informants ensured a rich mix of experience and perspectives. At the national level: one policy maker at the National Assembly, seven key officers/managers (five in Ministry of Health (MOH) and two in Ministry of Culture, Sport and Tourism),  two local non-government organisation representative and two international development partners. At the local level: 32 staff working in the health system from commune health station, district hospital to provincial health department, 1 project officer, and 12 representatives at two communes (including local women’s union, local leader and village leaders). Gender balance was 29 females and 28 males, and distribution in term of professional background included sixteen doctors, fourteen nurses/midwives, ten public health professionals, and seventeen others including social sciences, social work, and administration. Forty informants worked within the health system and  seventeen working in the related DV support services. The aim was to learn from people at all levels of the implementation process, from leading national policy makers and influencers (e.g. from the National Assembly, senior department authorities, NGOs), through the provincial policy makers and program managers, to clinicians and health workers in provincial, district and commune health services.

### Guiding questions for interviews

The questions in the interview guideline were developed based on Walt and Gilson’s policy triangle, which places the actors at the centre of three main factors: content, context, and process [9]. This model has been widely used and found to be effective in health policy analysis [6, 10, 11]. Open-ended questions were added during data collection depending on the stories and knowledge shared by the informants. Some factors that influence implementation of DVPC law within the health system were identified as themes from the literature review and from consideration of the key elements of existing models for health systems designed to address violence against women, including direct service delivery, human resources, financing, coordination, leadership and governance [12, 13].

### Position of the researchers and approach to accessing participants

A research team should mix ‘insider’ and ‘outsider’ collaborators to enable comprehensive understanding of the policy evolution [7]. Thi Minh Le works in a university and can be considered as being an “outsider” observer of the DVPC policy process. However, she also plays the role as an “insider” as she has extensive professional experience within the Vietnamese health sector. In order to see the complex policy dynamic and revolution and to access to the key informants, three field site coordinators in Hanoi and two selected provinces were invited to explain the objectives of the study, to advocate for the project locally and to introduce participants and arrange suitable times for data collection. The first author approached policy actors at the national level through introductions by the field coordinator who worked in the Ministry of Health. Once key informants were contacted and interviewed, other participants were identified via the snow ball technique. All participants in the two provinces were approached via introductions with the field coordinator who worked in the provincial health department. All IDIs and FGDs were conducted by the first author, based on the semi-structured interview guidelines.

### Data collection

#### Primary data

Data collection was conducted in Hanoi (national level) first to gain an overview of the policy process. This was followed by fieldwork in Hai Duong and Bac Giang province. Written informed consent was obtained before interviews and FGDs. Interviews were conducted either in offices or meeting rooms of the organization where participants worked, or occasionally at participant’s home at their preference. One interview was conducted via skype because the informant was abroad. The length of the interviews ranged from 40 to 120 min.

FGDs were conducted in meeting rooms of the commune health centres. One trained researcher from Hanoi University of Public Health assisted with the FGDs. The average length of FGD was about 60 min.

All interviews and FGDs were conducted in Vietnamese. Interviews were digitally recorded and transcribed. One key informant requested not to be recorded, and contemporaneous written notes were taken during the interview.

#### Secondary data sources

Review of 63 documents related to the main law on DV prevention and control was conducted. These documents included reports, policy documents, training materials and journal articles in English or Vietnamese, published between 2003 and 2018.

Secondary data pertaining to DV in Vietnam nationally and in the selected provinces (between 2009 and 2017) were collected and reviewed. The source of the data was the Department of Families, within the Ministry of Culture, Sport and Tourism (MOCST). This is the key coordinating government actor for implementing DVPC Law in Vietnam. The research team gained formal approval from the deputy head of the Department of Families that allowed access to secondary data for research purposes. The data were gathered via the administration information system in MOCST (according to the guidelines of the Decree 23/2011/MOCST dated 30/12/2011). Local authorities at the commune level are responsible to report DV cases in their communities on template forms periodically, and then send the reports to the district and provincial levels. This data includes aggregated data from the health system. The Department of Families (MOCST) collates the data from all provinces to obtain national level data tables that are related to DVPC implementation. The Department of Families (MOCST) prepared the data before sending to the research team.

### Data analysis

#### Primary data

NVivo 10 and Mindjet software were used to analyse the data. To generate the initial codes, the research team commenced with themes based on Walt and Gilson’s policy triangle and the research objectives [9]. According to this model, all themes (process, content, context and actors) were reviewed and analysed. Policy processes (agenda-setting, development and implementation with the focus on health system) are the main focus of the current paper. The *policy content* may lead to the different responses of actors and may effect on the health system. Different *policy actors* were mapped. Involvement of actors can be influenced by their agendas, mechanism and powers. The *wider policy context* such as political, socio-cultural, international context, and organizational/governance context can be either enabling or barriers factors. Themes were reviewed and refined after initial coding. Agenda setting was coded based on Kingdon’s theory of multiple streams [14]. Results were extracted and mapped based on relevant codes and themes. Data were analysed in Vietnamese language and the codes and report were written in English. Selected illustrative verbatim quotes from Vietnamese transcripts were translated into English.

#### Secondary data

Policy documents and reports were reviewed using a policy proforma to summarise the content of the documents. The content of different policy documents was compared. For example, the initial definitions of DV in the draft DVPC Law were different with international definitions of DV.

Secondary data from administration information systems (in Vietnam nationally and in two provinces) were reviewed and analysed. However, it became immediately apparent that the quality of data was weak, with massive under-reporting of incident cases of DV: the rate of households with DV incidents recorded nationally from 2012 to 2017 ranged from 0.057 to 0.14%. The total number of DV cases in 2017 in Bac Giang and Hai Duong province was 259 and 78 cases respectively, which lack face validity when compared to much higher estimates in self-report surveys with women in community settings in Vietnam [15]. As a result, only limited indicators relating to health services have been presented in the findings.

### Ethics approval

This study obtained approval from the Human Research Ethics Committees at Queensland University of Technology (Decision number 1700000703/QUT) and Hanoi University of Public Health (Decision number 345/2017/YTCC-HD3 dated 31/8/2017).

## Results

### Overview of DVPC Law development and implementation process

The DVPC Law was initiated in 2003 and developed by the Social Affairs Committee of the National Assembly. The law was developed between 2005 and 2007 with the aim of preventing domestic violence in Vietnam, especially violence against women. This Law was passed by the XII National Assembly of the Socialist Republic of Vietnam at its 2nd plenary session on November 2nd, 2007. This Law came into effect from the first of July 2008.

On May 2008, Directive No. 16/2008/CT-TTg became one of the first documents guiding the implementation of the DVPC Law. According to this document, the implementation guidelines were to be developed by a committee coordinated by the MOCST. The MOH also participated in the development of the implementation guidelines within the health system. The MOH approved the guidelines (Circular 16/2009) in 2009 and updated the contents in 2017 (Circular 24/2017).

### The policy process surrounding the law on domestic violence prevention and control (DVPC) in Vietnam

The evolution and implementation of the DVPC Law is described in two parts: 1) policy development (2003–2007), 2) policy adoption (2008–2009) and 3) implementation within the health system (2010–2018).

### 2003–2007: agenda setting and the development process in the context of the Millennium Development Goals

#### Agenda setting (2003–2007): the DVPC Law was galvanized by three streams

The first stream is the problem stream. DV received public attention through various civil society organizations, supported by the government led newspapers and national television channels. Research on DV was conducted in order to raise awareness of the public on this issued, primarily with the support of NGOs and civil society organisations in Vietnam, and financial and technical support from development partners such as UNFPA. In addition, behaviour change communication activities were carried out (UNFPA project), targeting the general public through the existing network of governmental organisations, NGOs and mass organizations. The mass media was active in raising public awareness with support from UN agencies, particularly after revelation of a number of severe cases and tragic stories. Local NGOs together with social pressure from the media and women’s organisations advocated for action by the government. These activities led to significant increases in awareness among policy makers, resulting in DV being introduced into the National Assembly’s law formulation program.

The second stream is the policy stream: although the specific focus of this paper is on DVPC Law since 2003, the policy was preceded by several decades of activities. In 1980, Vietnam signed the Convention on the Elimination of all Forms of Discrimination against Women (CEDAW) and ratified the convention 2 years later. In 1994, Vietnamese delegates attended the International conference on Population and Development, which resulted in gender equality becoming an essential component of policymaking and implementation in the area of reproductive health. In 1999, as a member of the United Nations, Vietnam participated in a workshop to set up the MDGs commitments, which included a goal and multiple targets and indicators related to gender equality. Once the commitments were made, Vietnam received considerable support from international organizations including United Nation agencies and international non-government organisations to achieve the goals. Policy designed to promote gender equality included the Gender Equality Law and Vietnam’s development targets within the Millennium Declaration that address the need to reduce women’s vulnerability to DV. Decision 106 in May 2005 was signed by the Prime Minister ratifying the Vietnam Strategy on the Family, which sets targets to reduce DV in the community. As a result, DVPC Law needed to be developed.

The third key stream is the political stream. Commitment of the Government to the international convention such as ICPD and MDGs raised the profile of efforts to reduce women’s vulnerability to DV and underscored the need for the Government to act. Putting DVPC law formulation on the political agenda was one of the major plans of that the Committee of Social Affair of the National Assembly for their term.

**Fig. 1:**
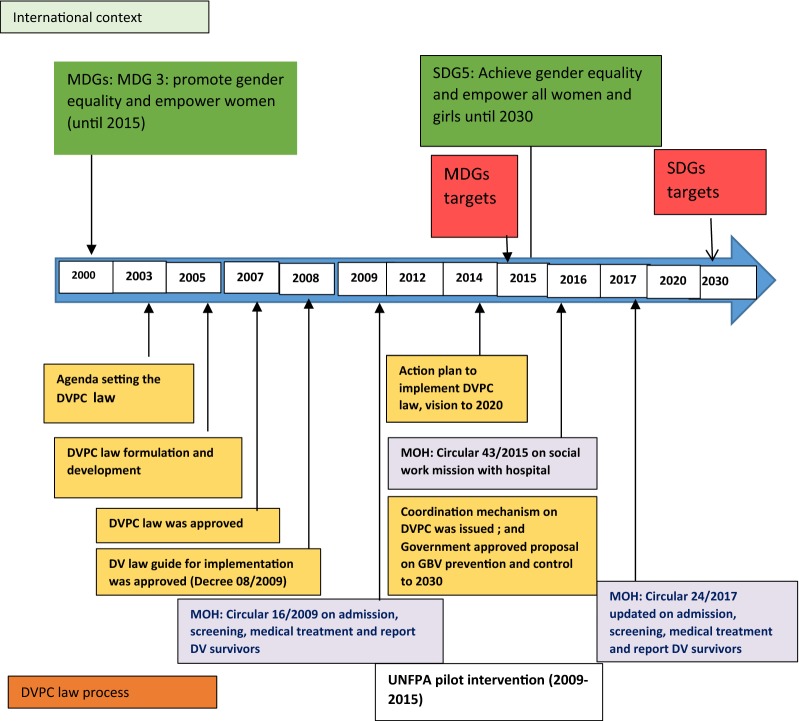
The DVPC Law evolution process in Vietnam

Therefore, a window of opportunity opened which facilitated DVPC policy development [14]. In the 9th National Assembly session in November 2005, after several years in the agenda setting stage, the Social Affairs Committee finally submitted to the National Assembly a proposal to develop the DVPC Law. By November 19th, 2005, the National Assembly assigned the Social Affairs Committee responsibility for developing and submitting a law on DVPC (Fig. 1).

#### Development (2005–2007): development process was quick

After 2 years of agenda setting, in the ninth National Assembly session in November 2005, the National Assembly submitted a proposal to develop the DVPC Law. In implementing the National Assembly resolution on the development of laws and ordinance No. 49/2005/QH11, dated November 19, 2005, the Committee of Social Affairs of the National Assembly was instructed to develop a law on DVPC. The Drafting Committee for the DVPC Law was under the direction of the Social Affairs Committee and included two units. One was the Drafting board and the other was the Editing committee to assist the Drafting board in collecting information, editing and writing the draft law. The content of the draft law took into account international standards on DV legislation and experiences learned from intervention projects and other countries during law development.

The consultation process for the development of DVPC Law included an advocacy workshop, online comments via a website, and technical meetings. The workshop included participants from different national and provincial organizations in different sectors. While citizens in the community were not directly involved in the policy process, they were indirectly represented through community leaders such as the chairperson of the People’s Committee or through website forums. Some NGOs and UN agencies sent their comments, research results or pilot intervention projects directly to the National Assembly to serve as references in the development of the law and implementation guidelines.

After three major consultation workshops in the three main geographic regions in Vietnam, the gender-neutral concept of domestic violence was adopted, instead of acknowledging that the key issue is violence against women. The roles of different actors were clarified and listed, and agreement was reached on health insurance coverage.

The Law was passed by the XII National Assembly of the Socialist Republic of Vietnam at its 2nd plenary session on November 21, 2007, and came into effect on July, 1 2008 [16]. The approval of this law demonstrate significant advances by the Vietnamese government toward ensuring gender equality and protecting rights of the people, especially women.

### Adoption and Implementation process (2008–2018)

#### Adoption: A comprehensive legal framework was established continuously to enable environment to implement DVPC

The development of DVPC Law in 2007 created a legal framework and enabling environment for implement DVPC. To facilitate the implementation of the DVPC Law, the government issued various decrees, circulars and national plans of action that outline the roles and responsibilities for implementation, monitoring, reporting, coordination and budgeting of line ministries, people’s committees, mass organizations, communities and individuals. In practice, the adoption and implementation process of DVPC Law happens continuously and unending. The DVPC Law is a multi-disciplinary law. In accordance with the DVPC Law, the Department of Families MOCST is the agency required to manage the DVPC Law. This actor developed guidelines for the implementation. Other actors involved in the development process included the MOH, Ministry of Justice, and Ministry of Information and Communication. On 22/9/2009, the MOH approved the Circular 16/2009 on reception, medical care and reporting of DV survivors at clinics and health institution with the supports from UNFPA. This Circular was revised on 2017. This is the platform for implementation of DVPC Law within the health system.

#### Implementation (2010–2018): A slow process within the health system

If the policy implementation process is divided into 4 stages (preparation/awareness, adoption/pilot, integration and maintenance) the evidence at present (2019) suggests that the DVPC implementation process within the health system in Vietnam is still at an early stage of integration. In practice, the implementation of DVPC Law within the health system was slow and key milestones were delayed. The health system only started to implement Circular 16/2009 in 2012 (3 years after it was issues). There was only partial implementation in 24/63 provinces from 2012 to 2015, and full implementation was not achieved by 2017 (MOH and MOCST report 2018).

##### Period 2010–2012

Challenges for implementation were predicted early. In late 2009, MOH arranged three regional seminars to allow representatives of provincial health departments to discuss the implications if the guidelines. Challenges were identified, mainly because the guidelines were seen to affect all parts of the healthcare system in terms of service delivery and human resources. Limited resources for health services and inadequate health insurance coverage for DV survivors/victims were also major barriers. Most participants from provincial health departments perceived health services for DV survivors as impractical and unfeasible (MOH workshop report, 2009). As this MOH policy makers said:

“*Many challenges to implement. We realised that the integration of DV to the health system would be impractical and unfeasible from the development stage. In practice, no health provider was trained to work with DV victims and we need support from others system. The health system cannot comply strictly with the guidelines because of limited resources, capacity and infrastructure*”

In this period, the health system was responsible for care and treatment those DV victims/survivors referred from social protection centres and reliable addresses in community, mainly those with severe injuries, or DV survivors who directly accessed the health care facilities. Besides, international donors and organizations funded small-scale pilot projects as DVPC initiatives, providing DVPC services and interventions in selected provinces (such as Ben Tre, Phu Tho, and Nghe An province). The key components of intervention pilot project were to increase the participants of multiple sectors, coordination and accountability [17]. Some of evidences from these initiative was addressed in updating regulation of MOCST in guidance the implementation such as coordination mechanism (developed by MOCST 2013).

##### Period 2013–2015

By the end of 2015, only 24 out of 63 provinces had reports on the implementation of DVPC within the health system. The data was poor and under-reported, capturing only 15,573 cases that accessed the health services over the 5 year period (2009–2014). And according to the report of the evaluation workshop in 2014 conducted by MOH, the main barriers of implementation of the DVPC regulation were the health system lack of readiness to integrate DVPC into their service model, a lack of budget for DVPC implementation, a lack of trained practitioners capable of responding to DV, and the fact that the DVPC regulation was not obligated, therefore, no reward or sanction was applied. A project officer stated:

“*The Circular 16 is optional, in theory, all health institutions have to adopt and adhere. In practice, we know that only provinces with intervention project funded by NGOs or international agencies implemented the regulation such as training, providing services and referral. Several provinces without fund only provide basic care and treatment services. In many provinces, health managers even did not know the existence of the DVPC regulation, or they knew but did not apply it*”

A health manager at the district hospital said:

“*There is no incentive or sanction of implementation DVPC. If we did well, there is no reward. If we did not do, nothing happened*”.

At the same time, UNFPA piloted second round intervention in Ben Tre and expanded to Hai Duong province (2012–2015), piloted the “minimal intervention package” in order to response to DV. In this model, the response within the heath care consists of selected screening DV survivors, providing treatment, referring to other supported services if needed and collecting/reporting data. The project was successful locally, but it was not scaled up throughout Hai Duong province and nationwide due to insufficient resources. A health manager said:

*“Even maintenance of pilot the DVPC activities is difficult because of limited budget. For example, DVPC leaflets were run out, new recruited health providers were not trained and the software for entry data of DV was died. The provincial government approved the plan to scale up the pilot project to the whole province as commitment with donor. However, as far as I know, only 2 pilot district hospitals did DVPC supported services and report data”*

A health provider at district hospital shared his de-motivation of the DVPC implementation after the end of pilot project:

*“The provincial health department even stated that it is not necessary to submit annual DV data for them, but we should submit data directly to the provincial department of culture for synthesis”*

Before 2015, the annual state budget for DV prevention and control was mainly dependent upon external donor allocations (in selected provinces only) and provincial budgets (if any). After 2015, the DV program was subsumed under the national program on gender equality. However, even with this change, the budget in reality was much lower than initially promised (e.g. the DV budget was reduced by 20% in 2013 and a further 50% in 2014 because of slow development of the economy) [18]. Therefore the implementation of the DVPC became short-term focused, rather than being a long term process. Due to limited resources for DVPC within the health system, the activities noted above were primarily funded by UNFPA. However, fund from UNFPA is often considered as “*temporary project*”, therefore, as UNFPA staff stated that:

*“We expected to strengthen the health care system. We hoped our official development assistance fund would be used similarly as an extra government’s resource allocation. However, health managers often considered our money would be used as a temporary specific project. The activities only run when the project invested money, these activities would ended when the project ceased”*.

##### Period 2016–2018

By 2017, 10 years after the DVPC Law was issued, not all provincial health systems had fully implemented DV regulations. By 5/2018 (the end of data collection), both provinces in this study still followed the instructions of the out-dated Circular 16/2009, due to a lack of specific guidance on implementing of the recent circular. An project officer shared:

*“I think health system has not considered DV as a problem. There are too many things related to disease treatment and prevention to deal with and to spend money. Although health managers aware that they should adhere the DVPC Law, the main problem was they had not considered DVPC as a part of public health. Health managers were awared of the new Circular on DVPC, however, asking hospitals to implement without clear and specific guidance is difficult. For example: who should screen the DV survivors: an administrator, a nurse or a doctor; who should provide counselling and reporting: new hospital social worker staffs (note: should be recruited from 2015 as new regulation) or doctors again. Many district hospitals even have not established social work unit. Therefore, I thought it was very difficult to translate the regulation into action”*

### Content

The DVPC Law in Vietnam adopted a local definition of DV as the focus of the legislation, rather than the generic UN definition of violence against women in MDGs (2000) and SDGs (2015) [8]. The UN definition includes explicit reference to violence against women and gender based violence, in recognition of the gendered nature of this crime, and the UN SDG5 refers explicitly to ‘*eliminating all forms of violence against all women and girls*’ [8]. However, this gendered construction was excluded from the conceptualisation of DV adopted the official definition of gender-based violence in the DVPC policy in Vietnam [19]. In Vietnam, according to the minutes of a commentary meeting on DVPC Law development, many national assembly members argued that the victims/survivors of violence are not only women and girls. Policy makers suggested that the gender specific term “violence against women” should be modified to be gender neutral, in order to imbed recognition of violence against both women and men within intimate relationships. As a result, the term “domestic violence” was adopted for policy documents [16, 20]. The rationale provided was that the terminology ‘domestic violence’ can include both violence against women and men within the family, and violence against the elderly.

Although the official position was to reduce emphasis on the gendered nature of DV, it is important to note that interviews with most key actors in this study suggested that the main objective of the law on DVPC is still primarily designed to protect women, who are over-represented as victims and survivors of domestic violence. A representative of NGO stated:

“*The Law targets all family members as some members of the National Assembly argued that men, children and elderly people may experience some forms of domestic violence. Evidence of this was found in secondary data and baseline data. However, the Law’s main objective is protecting women as the largest population of domestic violence survivors*”

#### Localised versus standardised definitions of DV

The ways in which DV is understood and defined have implications for how the law was developed and is implemented in Vietnam (See Additional file 1: Table S1 shows concept framing of ‘domestic violence’ and four related types of violence, in Vietnam’s official context). While the UN defined violence against women, Vietnam government purposively selected DV concept in developing DVPC Law. The UN definition was not officially used in the content of the DVPC Law because the term DV and sexual violence (within the family) were inclusive and de-gendered. Furthermore, DV in Vietnam was defined in more narrow terms listing 4 types (physical, emotional, sexual and economic violence) with 9 specific acts of violence. The definition of sexual violence is particularly narrow, with reference made only to ‘forced sex’ within marriage to describe sexual components of domestic violence [16].

Similarly, the focus on physical violence acts in Vietnam is relatively narrow and tends to be consequence-oriented. In Vietnam, the terms indicate that only those survivors who contract “injuries” or “evidenced-based symptoms” as a result of assaults are truly victimised and deserving of receiving support from health services and welfare systems.

The DVPC Law stipulates that *“all expenses for medical care and treatment for domestic violence victims shall be covered by the medical insurance funds if victims hold medical insurance cards*” [21]. This is one of the more recent elements of the law, and was added to improve the legal and financial protection of DV survivors who are covered by the health insurance scheme. Prior to 2008, DV was considered as “*intentional injury*” and therefore not covered by health insurance as the obligation for costs was presumed to reside with the perpetrator [22]. The DV survivors could not receive treatment covered by health insurance if they informed their carers that the “*injury [was] caused by intentional acts*”. From November, 2008, the health insurance law was changed to reflect the new DVPC Law, with the aim to better meet the needs of both clients and health providers [21].

#### Impact of the content law to poor implementation

The development of DVPC Law in 2007 created a comprehensive legal framework and enabling environment for DV program implementation. Arguably however, it is impossible to implement a policy that is defective in its conception. A good policy should be formulated based on evidence and international standards. Without proper standards, a policy may proceed in unexpected directions. For example, the data from the DV reporting system in Vietnam was not sufficient to be used to assess MDG3 achievement in terms of violence against women, and indeed, cannot be used for comparisons with other countries. In Vietnam, this problem is reflected in the lack of indicators on violence against women in the national report on MDG achievements. Only one academic paper was able to illustrate DV achievement of MDG3 in Vietnam in terms of attitudes about DV among women, using the Multiple Indicator Cluster Survey (2006–2011) [23].

There are significant gaps between the content of DVPC policies and its implementation within the health system. DV survivors face multiple challenges when accessing health care services. One of the key issues is the lack of knowledge and skills of health workers. According to the interview data, health workers generally do not receive training on violence screening and counselling (except for those in the selected province involved in the UNFPA intervention project during 2012–2015).

“*All health workers in the selected district hospital and commune health station attended 2*-*day training on DV and how to response to DV survivors. The project later, applied to representatives of other district in Hai Duong*. *We think without project, there is no training for health workers in other provinces”*

In contrast, there was no training on DVPC for health workers in Bac Giang province. A health manager in Bac Giang stated:

*“We did not receive training on implementing the DVPC Law and the circular 16/2009. The guidance was still general. Implementation without clear guidance is challenged and costly.”*

Health professionals usually treat DV survivors as “normal patients” with “injury” when deciding on their treatment and follow up care. Health workers interviewed in this study indicated that they usually skip screening and reporting violence unless women or men declare it to them and ask to have it reported.

*“If the patient did not declare, we did not know. Rarely, patients reported themselves as domestic violence survivors. Anyway, we all treat them as patients with injury”*

Another barrier to implementation is the lack of qualified social work staff. There are very few social workers in the Vietnamese health system who have specific responsibility to support DV survivors. Social work units within provincial hospitals were required to be established since 2016–2017 (according to Circular 43/2015/BYT). Typically, these units are tasked with counselling patients and family members, providing social supports and charity. However, in many locations, it is clear that Circular 43/2015 on social work within district hospitals had not been implemented by May 2018. Women who experience DV and who accessed health care services usually are not offered counselling by social workers or other appropriately trained health professionals, and there is little or no follow-up when women leave hospital. One health manager in the district hospital commented:

“A s*ocial work unit was only established in the provincial hospital. Until now, we have not had social work unit or any social worker*”

Although there is some health insurance coverage for DV care and services, DV survivors still have to pay 20% to 100% of the cost of treatment depending on availability of health insurance card, and the level of insurance cover they may have. Moreover, according to Circular 93/2012 and Circular 243/2016 from the Ministry of Finance, patients affected DV and other forms of violence are required to pay an out-of-pocket payment from 1,150,000-2,882,000VND (50–120 USD) to obtain a certificate of infirmity. This paper is a necessary piece of evidence to pursue legal processes [24, 25]. (Note: the cost is only covered by State’s budget if there is a police letter requesting for forensic examination). As a consequence, survivors without a health insurance card may face a double crisis resulting from the consequences of DV and the cost of treatment.

From 2016 to 2017, after receiving feedback from a pilot project and from data on DV from various hospitals, the MOH revised and replaced Circular 16/2009 with Circular 24/2017, simplifying the guidelines for implementation of DVPC Law within the health system. The main revisions included:

- Simplifying the form used for record DV (reducing a 4-page form to 1 page);
- Provision of guidance for health workers to report multiple incidents and combinations of DV experiences (e.g. physical, emotional, sexual, and economic violence) instead of only reporting the most severe form of DV (most often where there was clear physical injury);
- Reducing the frequency of the DV report from 6-monthly to an annual report, to reduce workload; and,
- Introducing the provision that hospitals should arrange temporary shelter for DV survivors for up to 24 h in an establishment within the health care system. After 24 h, it is now recommended that survivors are referred to other organizations in the community for further social support.

These revision were based on the feedback of health care providers on challenges of implementation. However, by 5/2018, both study provinces still implemented the previous (out of date) Circular due to waiting for the detailed instructions on implementation.

### The actors: multiple actors involved in the DVPC process, however, the coordination was weak

As DV is a crosscutting issue, it was developed by the Committee of Social Affairs of the National Assembly, rather than a specific Ministry. Actors involved in the policy development included related ministries, provincial authorities, international development partners (mainly UNFPA, WHO), and civil society groups. Table 1 mapped the different actors involvement in the developing and implementation process.

**Table 1:** Actors’ involvement in the domestic violence prevention and control law process in Vietnam

Level	Actors	Agenda setting	Development phase	National DV survey	Administration	Implementation phase
National level	*National Assembly*	*x*	*x*			
	Vietnam Population and Family Planning committee^a^ (VPFPC)	x	x			
	*Ministry of Health (MOH)*	*x*	*x*		*x*	*x*
	*Department of Family, Ministry of Culture, Sport and Tourism (MOCST)*		x		x (coordination)	x
	Ministry of Justice	x	x		x	x
	Ministry of Labor Invalid and Social Affair	x	x		x	x (in social welfare service system)
	Police, Court	x	x		x	x
	Ministry of Information and Communication		x		x	x (in mass media)
	Ministry of Education and training				x	x (in education system)
	General Statistic Office (GSO)			x		
Local level	*Provincial health department and district hospital, *		*x*		*x*	*x*
	*Commune health center*					*x*
	*Provincial, district and commune People committee*		*x*	*x (some selected province)*	*x*	*x*
	Police, Courts		x		x	x
	Provincial department of Culture (coordinator)				x	x
UN agencies	*UNFPA*	*x*	*x*	*x*	*x*	*x (some small scale projects)*
	WHO	x	x			
Development partners	Danish Embassy,	x	x			
	Swiss Embassy	x	x			
	Spanish agency for international development and MDG achievement fund.			x (funded)		
Civil society organisations	*NGOs,*/international NGOs		x	x		x (some small projects)
	*Vietnam women union/youth union/Vietnam father front*	*x*	*x*			*x*
	Mass media	x	x	x (dissemination workshop)	x	X
Users	Users (survivors, pepetrators, families and community)			x		x

^a^VPFPC was closed down in 2008, the department of Family from VPFPC was merged to MOCST

Italic indicates the actors involved in this study

It was the first time in Vietnam’s history that NGOs, researchers, and other specific organizations and institutions were invited to present their ideas about the Law in a public policy development process. In the overall process, UNFPA was the key international actor that financial and technical supported all stages of DVPC policy development and implementation. Other invited actors in this policy development process included civil society groups, other development partners, and local authorities at different levels.

Britto et al. [26] developed a model of coordination processes that represented the common dichotomy of vertical versus horizontal coordination processes. The vertical dimension refers to coordination within a typical sector such as a health system (national, provincial and local level) [26]. This contrasts with horizontal coordination across actors (health system and related sectors). Regarding DVPC Law implementation, the government applied mechanisms to coordinate across different actors for the provision of essential support for women subjected to DV. The DVPC Law needs cooperative horizontal implementation, but this is rendered difficult because the structure of decision making, budgeting and reporting in relevant ministries is vertical, from central to local administrative systems. Specifically regarding DV, each ministry is essentially responsible for one specialized issue. Respondents indicated that health workers felt embarrassed about the lack of cooperation with other sectors such as police or local authorities, and were concerned about how this impacted their capacity to provide support for DV survivors, beyond first aid. This situation exacerbates the limited supports available for women who have experienced violence. As one health worker in the district hospital commented:

“*We provided treatment for them (survivors), however, we do not know how and where to refer them after treatment*. *We provide medical care for patients and discharge them after treatment. We think they should go back to the community to seek other legal support*”

“*Our hospital do not have any psychological doctors or social workers. We can provide only physical care and treatment. If they (survivors) had depression symptom, we can prescribe sedative. We think they (survivors) can seek further social supports from women’s group after discharge from our hospital”*

The situation was best in a district hospital in Hai Duong province where UNFPA had implemented a pilot intervention. DV survivors were referred to the counselling room within the hospital. Health workers know the contact details of police and other support groups, however, the link between health workers with external actors to support DV survivors was limited due to time constraints and complicated processes. A health manager stated:

*“Sometimes, we receive DV cases sent from the police or women’s union representative. After providing a medical examination, we send DV survivor a certification of examination. If a survivor visits a health institution first, we usually send them to the counselling room (within hospital) after providing care and treatment. We rarely call the police because of time consuming and complicated processes”*

Due to the lack of funding and human resources for DVPC, implementation has effectively become optional, and therefore vulnerable to the vagaries of individual health services. Without explicit, strategic budget allocations, there have been many delays in the national and local DVPC systems in the health sector. Very few private institutions report data on screening DV survivors.

### The wider contexts that influence DVPC implementation

#### Political context

Political authorities, both nationally and locally in the study provinces were officially committed to and supported the development and administration of DVPC Law. This was evident in a number of policies after the renovation period that commenced in 1986. As the economy transitioned toward market model, Vietnam entered into trade agreements with other countries and the government is reforming national law to respond to its international commitments [27]. This legal reform includes human rights, gender and social protection [28].

In Vietnam, the National Assembly has played an active role in promoting the advancement of Vietnamese women through developing its legislative documents such as DVPC Law. As this officer of the international development partner stated:

*“The National Assembly pushed the agenda setting and issued the law on domestic violence prevention and control in Vietnam quickly”*

This officer also added: “*Deputy Prime Minister always supports gender equity initiative programs; he often attend[s] our activities and events on violence against women or SDG conference.”*

At the local provincial level, in both study provinces, there are numerous documents from the provincial people committee that give directions toward implementation of DVPC Law. Political commitment is essential for several reasons: (1) to ensure leadership commitment, (2) to generate effective inter-sectoral coordination and collaboration, and (3) to contribute to the process of changing awareness and behaviors among the general population. The health system was not unequivocally supportive of the DVPC Law immediately. However, the commitment to embrace DVPC Law implementation increased demonstrably once the law was issued.

#### International factors

The influence of global institutions and policy responses is important in shifting national government priorities [29]. The commitment of the Vietnam government to meeting international milestones such as CEDAW, MDGs and SDGs and is clearly evidence in documents [30] and through the interviews. A participant from NGOs shared:

Role of international organizations and global context in the policy is important to the policy process. As DV is one of the key indicators to monitor the achievement of MDG3 and SDG5 on gender equality, the UN in Vietnam and some international partners (such as Danish embassy and Spain Embassy) had prioritized efforts to address this issue and pushed the policy development quickly although there were concerning of its feasibility. A participant from NGO said:

*The fact that the Law was passed seemed to please international donors even though almost all understand that it is challenging to apply it in practice. The feasibility of the DVPC Law was questionable in the context of Vietnam from the beginning*”.

However, a policy maker did not agree as his comment:

*“The development process for the DVPC Law faces the challenge of changing the attitude of the community. Gender inequality in Vietnam has existed for thousands of years and it takes time for them to understand the problems. However, we think the DVPC Law is necessary for protecting the rights of women, as it is the first formal law on this issue. We did try to develop the DVPC Law, so that it can be put into practice by taking into account many comments from all over the country”*

UNFPA as the international donor/partner relevant to this study, influenced both the development process and the implementation of DVPC Law in Vietnam. UNFPA provided both financial and technical resource to pilot the DVPC model of minimal intervention package. In this case, UNFPA used financial resources to shape the implementation in selected provinces according to the project’s objectives and design. For example, in Hai Duong province, the implementation in the period of 2012–2015 was limited within the 2 districts out of 10 districts as the objectives/design of UNFPA project. And within the 2 districts, only 6 communes were selected for pilot intervention. The two district hospitals in Thanh Mien and Kinh Mon became focal points for referral all victims/survivors in the whole province.

#### Organizational/governance context: decentralization and financial autonomy resulted in delayed implementation within the health system at the local level

A key purpose of decentralization is to eliminate unnecessary layers of bureaucracy, untangle chains of command, and link greater percentages of fiscal and human resources. With decentralization, organizations and health managers have high levels of autonomy, local governments at provinces can have space for autonomous decision making [31, 32]. Health financing in Vietnam has been decentralised and the state budget for health is allocated through the central budget and local budgets.

A particular barrier for implementation is that there is no budget line within the health system specifically for DVPC. Because of decentralization in decision making and budgeting throughout Vietnam government agencies, is it up to each health institution to allocate funding for DV from their existing budgets. The level of funding depends on the interests of particular health managers on DVPC and advocacy for DVPC within the province. Without budget allocation for DVPC, the implementation was delayed. In the context of financial autonomy in the Vietnamese health system, health managers who participated in this study indicated that they prioritized budgets for disease treatment and improving hospital quality rather than DVPC. As a health officer in the MOH stated:

“*There is no budget for implementing DVPC*, *health managers in local hospitals often prioritise big and important issues such as treatment and quality assurance rather than DV”*

The budget allocation for district hospital is based on the number of patient’s bed. A health manager in Bac Giang did not mentioned any reason of not allocating fund for DVPC directly but stated the challenges of managing low budgetary resource:

“*The annual budget allocation is limited and often estimated based on the number of patient’s beds. We had to spend for salary, medicine, infrastructure and many other things. We have not mentioned numbers of our medical expenses were not refundable accumulately because the health insurance agencies rejected expenditures.*”

#### Socio-cultural context

Debates during the development process focused on the definitions of DV and the broad term ‘violence against women’ (as reflected in the title of the law); concerns about perceived breakdown of traditional family structures; the need for legal supports for survivors; the roles of different actors in providing services; and the feasibility of the law in practice. Many issues were contentious and contested, as this quote from a policy maker captures:

“*We faced quite a number of debates on whether the Law on DVPC would lead to a breakdown of the family, a rise in the divorce rate because some delegates think that the Law would interfere in the environment of the family. Once a woman leaves her husband’s house, she may find it difficult to go back. It is considered part of the Asian cultural context that there is gender inequality. This law will result in more divorces and difficulties for the children.*”

Socio-cultural context also influenced implementation stage. Socio-cultural norms prevent DV victims/survivors from accessing support services, and reporting DV incidents. Gender and family norms in Vietnam tend to prioritize family harmony. For many people, family harmony is seen as a balance between Yin and Yang, and this may help to sustain male aggression and female submission [15]. As a result, women survivors and family members often hide occurrences of DV. One health provider at the commune level said:

*“Even though we suspected a women is a DV survivor, she insisted that she only fell”*

Socio-cultural norms lead DV survivors and community tolerance and hide the situation of violence, even with severe DV cases. One project officer shared:

*“That’s why many severe cases and even deadly, occurred but the data was rediculouos underexpected. There were many undetectable cases. For family reasons, they arrange themselves without inform to the police. If a woman moved to the shelter, who would take care her kids. If her husband was sent to the prison, who would care for her family, who earn money. The woman also thinks for her husband and for the family, does not want to send her husband to the court”*

In this socio-cultural environment, DV statistics under-report actual incidence of DV and the extent to which DV has resulted in visits to health services. The total number of DV survivors reported at all health care service for the period of 2012–2018 in Hai Duong province was only 68 cases, while in Bac Giang province was 231 cases (source unpublished data of MOCST 2018). Such estimate share totally implausible as the combined provincial population in these two provinces is approximately 3.5 million people.

## Discussion

This case study of DVPC Law and its implementation in the health sector in Vietnam illustrates the serious system-wide challenges that must be overcome to prevent and respond to violence against women, especially in low resource countries.

Symon and Luzth [33] point to three distinctive stages of DV policy evolution: 1) male privilege and the right to discipline, reflecting the denial of domestic violence, which results in the social supports doing little to protect DV victims/survivors; 2) the right to protect, reflecting the informed stage where coerced social change and legal documents are established to treat DV responsively; and 3) mature stage, reflecting collaborative empowerment that addresses DV through an evidenced based collaboration approach [33]. The present study shows the evolution of DVPC Law in Vietnam over 15 years (from 2003 to 2018) through the first two stages. The change from denial stage to informed stage was significantly influenced by international political factors. Agenda setting for DVPC Law is best illustrated the policy window, as three main streams came together.

The policy development stage was shaped by the international treaties and commitments. There are some positive outcomes. Developing the DVPC Law met international obligations after Vietnam signed the CEDAW and became obligated to achieve the MDGs. The DVPC Law in Vietnam was the first legislative step towards a solution for DV survivors, and it was considered an initial success by the women’s movement toward gender equality in Vietnam. Compared to previous closed policy, DVPC Law was considered to be the first “open” policy in which multiple actors including NGOs and international partners were involved in the formative processes, and this was seen as a positive step towards a legal basis to protect DV survivors in Vietnam.

The findings of this study clearly show differences between development and implementation in terms of the various actors’ relationships. DV is a complex issue requiring multi-sectoral responses. While the relationships among actors (Ministries, international development partners, NGOs) showed extensive horizontal interaction in the development process, the implementation stage shows fragmentation of the system, which diminishes capacity of the 4 systems to identify and support DV survivors. In each sector, for example, the health system actors mostly interact within the vertical system and are closed off from interaction with other important actors such as the local authorities, women’s groups and the police. Within the vertical health system, survivors are “patients”, and treated for injuries, while most health workers hesitate to seek cooperation from other sectors to further support for survivors.

The evolution of the policy shows that political commitment and international factors were key enabling factors, while sociocultural contexts, decentralization and financial autonomy were barriers that stymie the DVPC process. Political and international factors stimulated DVPC Law development quite quickly. However, in the implementation process, there were more barriers than enabling factors. Political commitment and scale up of DV model of implementation were not sufficient. Socio-cultural factors suppressed of DV survivors’ access to health care services, and strongly constrains disclosure to authorities. Both outcomes cause under-reporting of DV in official records. In addition, similar to other settings, decentralization and fiscal autonomy in Vietnam resulted in delay and slow provision of DVPC health care services [34]. Lack of interest among health system managers and deficient budget allocation for DVPC erode the capacity for sustainable responses for DV survivors in the health care system [12].

The prominence of UNFPA in this process had both positive and unintended adverse outcomes. The findings reflect three different mechanisms of donor influence on the implementation stage of the DVPC policy process: 1) financial support, 2) technical expertise, and 3) other inter-sectoral leverage. UNFPA used financial resources and technical support to shape the implementation nationally and in selected provinces. UNFPA also pushed the government on development of the Circular on coordination mechanism among sectors response to DVPC. However, while the potential positive effects of UNFPA’s power to motivate actors toward some target objectives, there were unintended adverse outcomes and concerns among local actors about the pilot intervention. For example, concerns were expressed about the sustainability of the project to after funding ceased. In practice, after 2016, successful lessons from two selected districts could not be scaled up to the whole province and to nationwide as was originally expected.

In the context of international pressure to develop a specific law on violence against women, the DVPC Law in Vietnam was formulated hastily, without comprehensive evidence base, and with limited experience from pilot interventions. The policy was formulated at a time when key actors had limited knowledge and capacity to respond to DV. While global trends framed the legal response to address violence against women in terms of human rights and gender equality, DVPC Law development in Vietnam removed this gendered focus, reframing the issue as violence within the family. The content of the current DVPC Law illustrates the failure of the draft policy committee in convincing and lobbying members of National Assembly to adopt the standard UN definitions on violence against women. International development partners and non-government organizations had advocated for gender equality and women’s rights, and pushed for a legal agenda that framed the law as violence against women, as is done in many other countries [34, 35]. These actors were unable to succeed in changing the attitudes of members of the National Assembly, and thus the definitions of DV acts and consequences remain quite restrictive. Unfortunately, DV was defined as a health (rather than human rights) issue; framed within biomedical concepts and therefore, medicalized, this has influenced the responsiveness of the whole system. The medicalization of DV promotes focus primarily on injury, or “evidence-based violence”, eschewing the broader nature of DV. In turn, this policy framework influences the understandings of health workers and people in the community regarding denial and non-discloser of DV, which probably results in low numbers of DV survivors accessing health care services (only severely injured survivors tended to access health care service). When women did present to health services for support, survivors were treated as ‘patients with an injury symptom’, which obscures evidence of violence.

The future for action on DVPC is uncertain. Vietnam has become a middle income country and in recent years, there has been a major decrease in investment by international aids, development donors and partners that previously had supported implementation of health initiatives, including DVPC. Change has also been profound inside the Vietnam government systems, where decentralization within the health system and economic austerity have influenced budget allocations for DVPC. As a consequence, the implementation process is not obligatory, and vulnerable to the vagaries of individual health service managers’ interests in gender inequality and violence, clinical staff have not been inclined to engage in screening and referral for DV survivors, and DV incidence has been significantly under-reported. In other words, despite considerable effort to develop DVPC Law to achieve gender equity goals and reduce risks of violence against women, implementation has to a large extent been thwarted by obstacles within the health system itself.

The study has some strengths and limitation. The study has proven useful for studying DVPC policy evolution from agenda setting to implementation. It is the investigation and exploration of a policy process thoroughly and deeply across 15 years. However, the case study design limits within Vietnam, which can not be generalised to other countries. Secondary data for 10-year implementation could not be presented due to weak quality. The case study only focused on development and implementation within the health system.

## Conclusion

In summary, there are significant gaps in translating government commitment to MDGs into DV policy development, and between policy development and implementation. The evolution of DVPC policy in Vietnam illustrates the pressured nature of the development process, especially in the health field. If these shortcomings are not addressed, Vietnam’s SDGs commitments to gender equity and violence prevention will not be achieved. Policy revision is essential, with progressive changes are needed in the conceptualization of DV to reflect international definitions, budget mobilization for DVPC, enhanced monitoring and evaluation, and improved coordination between the health system and other support services that are sensitive and responsive to the needs of DV survivors.

## Additional file

**Additional file 1: Table S1.** Comparison between definitions of Domestic Violence internationally, and in Vietnamese law.

## Data Availability

All data used and/or analysed during the current study are available from the corresponding author on reasonable request.

## Abbreviations

CEDAW: The Convention on the Elimination of all Forms of Discrimination Against Women
DV: Domestic violence
DVPC: Domestic violence prevention and control
FGDs: Focus Group Discussions
ICPD: International Conference on Population and Development
IDIs: Indepth interviews
MOH: Vietnam Ministry of Health
MDGs: Millennium Development Goals
MOCST: Ministry of Culture, Sport and Tourism
NGOs: Non-government organizations
SDGs: Sustainable Development Goals
UN: United Nation
UNFPA: United Nation for Family Planning Agency
WHO: World Health Organization
