# Supplementary material for: The evolution of domestic violence prevention and control in Vietnam from 2003 to 2018: a case study of policy development and implementation within the health system
Source: Int J Ment Health Syst. 2019 Jun 8;13:41. doi: 10.1186/s13033-019-0295-6 (PMC6555957; doi:10.1186/s13033-019-0295-6)
Supplement: Supplementary file 1 — Additional file 1: Table S1. Comparison between definitions of Domestic Violence internationally, and in Vietnamese law. [file 13033_2019_295_MOESM1_ESM.pdf]

Table S1: Comparison between definitions of Domestic Violence internationally, and in Vietnamese law

Definitions	United Nation	DVPC law in Vietnam (9/2007)
**Term using**	Gender based violence or Violence against women : “behaviour by an intimate partner or ex-partner that causes physical, sexual or psychological harm, including physical aggression, sexual coercion, psychological abuse and controlling behaviours”	Domestic violence: “purposeful acts of certain family members that cause or may possibly cause physical, mental or sexual injuries to other family members”.
**Physical violence**	“acts aimed at physically hurting the victim and include, but are not limited to, pushing, grabbing, twisting the arm, pulling the hair, slapping, kicking, biting or hitting with the fist or object, trying to strangle or suffocate, burning or scalding on purpose, or threatening or attacking with some sort of weapon, gun or knife”	“corporal beat up, ill-treating, torturing or other purposeful acts causing harm to one’s health and <may be> life threatening”
**Emotional violence**	Emotional violence includes a range of behaviours that encompass acts of emotional abuse and controlling behaviour.	“insulting or other intended acts meant to offend one’s human pride, honour and dignity and isolating, shunning or creating constant psychological pressure on other family members, causing serious consequences”
**Sexual violence**	any sort of harmful or unwanted sexual behaviour that is imposed on someone. It includes acts of abusive sexual contact, forced engagement in sexual acts, attempted or completed sexual acts without consent, incest, sexual harassment, etc. In intimate partner relationships, experiencing sexual violence is commonly defined as being forced to have sexual intercourse, having sexual intercourse out of fear for what the partner might do, and/or being forced to so something sexual that the woman considers humiliating or degrading	In DVPC law, the only sexual violence reference is limited to ‘forced sex’ within marriage and “forced child marriage, forced marriage or divorce and obstruction to voluntary and civilized marriage”.
**Economic violence**	Not available	Property damage or destruction, prevention of the exercise of legal rights and obligations, and forced work.
